# The effect of quiet eye training on golf putting performance in pressure situation

**DOI:** 10.1038/s41598-024-55716-z

**Published:** 2024-03-02

**Authors:** Qiao He, Yunzhou Liu, Yongtao Yang

**Affiliations:** 1 Sports Training Laboratory, China Institute of Sports Science, Beijing, 100061 China; 2 School of Strength and Conditioning Training, Beijing Sport University, Beijing, 100084 China; 3 Research Centre for Sports Psychology and Biomechanics, China Institute of Sports Science, Beijing, 100061 China; 4 Laboratory of Competitive Sport Psychological and Psychological Regulation, Tianjin University of Sport, Tianjin, 300381 China

**Keywords:** Quiet eye training, Golfer, Putting performance, Pressure situation, Sports psychology, Physiology, Psychology

## Abstract

To explores the effect and mechanism of quiet eye training on the accuracy of golfers´ putts in pressure situations and provides methods and basis for targeted attention training and control. 22 young golfers in China golf team aged from 13 to 18 were randomly assigned to the experimental group (quiet eye training group) and the control group (technical guidance group) according to gender. Both groups of participants underwent two consecutive weeks of push training (3 sets per day, 20 golf putts per set, rest for 3 min between sets) separately in accordance with the guidance of a professional psychological research group and an expert coach. Eye tracking technology, biofeedback technology, and subjective evaluation methods were used to test and analyze the push process of the two groups of participants before and after training under pressure situations (Eye movement behaviors and the heart rate were recorded by ASL Mobile Eye-XG and NeXus-2 biofeedback, pressure and state anxiety were evaluated by self-rating pressure scale and S-AI. Golf putting performance was recorded by a research graduate assistant). A higher hit ratio as well as lower pressure and SAI level was founded in quiet eye training group in the pressure situation, the quiet eye movement time and total fixation time was longer than technical group. The quiet eye training group has a better putting performance. Quiet eye training can improve the golf putting performance in pressure situations. After quiet eye training, the state anxiety decreased, the quiet eye movement time and the total fixation time increased in pressure situations.

## Introduction

The relationship between attention and movement performance is a research hotspot in the field of movement performance. For self-controlled closed form movement skills, excellent attention concentration and movement control abilities are often required^1,2^. Golf is the sport which needs a typical self—controlled closed form movement skill, in which requires a high degree of accuracy in the putting stroke and a high degree of attentional focus during competition^3^. However, in competitive pressure situations, athletes often experience heightened levels of anxiety, which in turn, adversely affects their attention and leads to a decrease in the accuracy of their putts. This phenomenon, known as over-pushing, significantly impacts their overall performance. How to effectively improve the attention control ability during putting has always been a problem that athletes and coaches are committed to solving, and it is also a key factor in improving sports performance.

Current research has shown that prolonged quiet eye can ensure athletes have sufficient time to process movements and avoid interference from irrelevant information^4^, and that prolonged gaze can help performers filter key information before completing their movement skills^5^. Chia et al.^6^ suggests that the quiet eye before the start of a movement is an important feature of a visual search strategy (quiet eye refers to a prolonged gaze or follow at a specific position before the movement occurs), and due to the dynamic complexity of the movement context, this visual search strategy can ensure that athletes have enough time to focus on task-relevant cues^7^. Other studies have shown that quiet eye is an effective indicator for evaluating attentional control. Prolonging the quiet eye period can reduce the adverse effects of competition anxiety, and quiet eye training is of great significant in combating anxiety^8–10^. In research studies, it has been found that athletes improve their sports performance by adopting more effective attentional strategies^11,12^. Additionally, quiet eye training has been shown to reduce the negative impact of anxiety and competitive pressure on performance, to a certain extent^13^. This type of training helps athletes better comprehend when and where to direct their attention towards different aspects of movement execution.

Quiet eye training is a psychological skill training method that enhances athletic performance by training athletes to master the quiet eye pattern which expert athletes use to accomplish a certain movement task^8–10^. Longer periods of quiet eye can be trained, and Broadbent et al.^10^ suggest that quiet eye is simpler and easier for athletes to master than other perceptual skills, as well as being easier to train. Gaze is a perceptual choice and quiet eye as a special behavior in visual gaze strategies, has attracted much attention^14^. The current main statistical indicators include: quiet eye movement time, which refers to the long-lasting gaze time of the action executor on a specific location before the action is initiated; the total number of gaze points, which refers to the number of gaze points that appear within a period or within a target Area of interest (AOI); the total fixation time indicates the sum of the duration of all fixation points falling within the interest area^15^. Precise control of these commonly used indicators is an important variable in improving motor performance through quiet eye training.

The current research on quiet eye training mainly focuses on the relationship between quiet eye and sport performance, while a lack of in-depth research on the impact of task types, project differences, action operating environments, etc., as well as the mechanism by which quiet eye training improve sports performance^16–18^. However, the emergence of lightweight portable eye-tracking devices has provided the possibility for researchers to explore attentional behavior in visually dominant movement skills in a more natural setting.

In view of this, the present study is intended to implement targeted quiet eye training for golfers to improve their putting accuracy under pressure situations and attempts to explore the mechanism of its movement with the help of eye movement technology, so as to provide a methodology and basis for targeted attentional training and control. This study will not only help to improve golfers' putting performance, but also provide a method to regulate the competition psychology of closed sports that require visual dominance, which will have important theoretical significance and application value. The hypothesis of this experiment is that after undergoing quiet eye training, the participants will experience an increase in their hit rate when putting under pressure. Additionally, their self-rated pressure level and state anxiety are expected to decrease while their quiet eye movement time and total fixation time will increase.

## Methods

### Participants

There were 22 athletes (12 males and 10 females) from the National Golf Junior Team, aged 13–18 years old, with 5–8 years of training. All participants were not color blindness or color weakness, had normal vision, volunteered to participate in the experiment and had not taken drugs or alcoholic beverages within 48 h before the test, and were paid appropriately after the experiment. All participants were informed of the protocol and purpose of the research as well as the associated risks and signed the informed consent to participate in the study. And they agreed publication of identifying information/images in an online open-access publication.

### Ethical approval

The study was conducted according to the requirements of the Declaration of Helsinki (2013) and was approved and followed the guidelines stated by the Ethics Committee of China Institute of Sport Science (2023081702).

### Study design

Mixed design. The group was an inter group variable, and the testing time was an intra group variable. The group included two levels: the quiet eye training group and the technical guidance group. The testing time included two levels before and after training. The dependent variables included hit rate, self-assessment of pressure level, state anxiety, heart rate, stationary eye movement time, total number of fixation points, total fixation time, and other indicators during putting under pressure.

### Procedures

**Figure 1:**
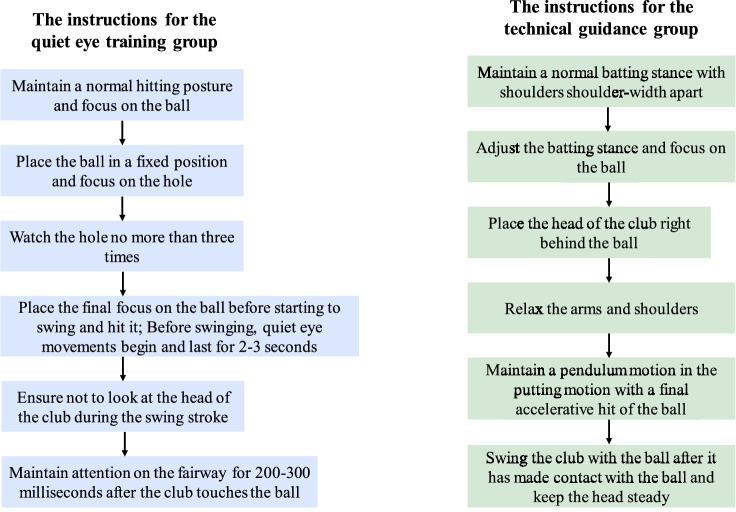
Instructions delivered to both groups during the 2-week training intervention.

The participants were randomly divided into two groups after matching according to gender, namely the quiet eye training group and the technical training group. Both groups of participants were required to practice putting for two consecutive weeks in accordance with the instructions (3 sets per day, 20 golf putts per set, rest for 3 min between sets) as shown in Fig. 1.

**Figure 2:**
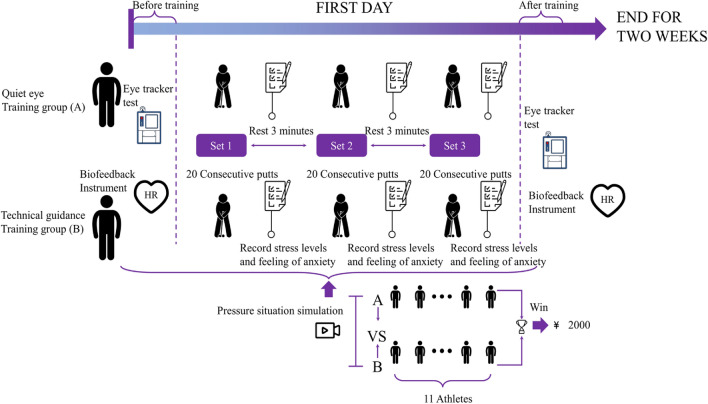
Pipeline of entire experiments.

As shown in Fig. 2, a portable eye tracker was used to test the gaze behavior of the two groups of participants during putting under pressure situations before and after training, and their heart rates were recorded synchronously by using a biofeedback device. At the end of each test, the participants were asked to quickly record the level of pressure (using a 10-point scale, where "0" means no pressure at all, "9" means very much pressure, and from 0 to 9 means gradually increasing pressure) and anxiety during the test. All participants putted at a fixed point on the green (3 yards away), wearing a portable eye-tracking device and a biofeedback device before the test, and entered the formal test after completing 10 putting exercises. At the beginning of the formal test, the eye-tracking device was recalibrated, and the participants were asked to complete 20 consecutive putts after simulating a pressure situation.

Pressure situation simulation: referring to the commonly used methods of simulating pressure^14,15^, the participants were told to participate in a putt test competition and score based on the total score of the group. The winning group will receive a team reward of ¥ 2000, and each person's score will directly affect the performance of their peers. The entire testing process will be filmed, and the results will be fed back to the coaching team.

### Experimental tools

Eye-tracker: American ASL Mobile Eye-XG portable eye-tracker, including two parts of the components, monocular corneal reflex system to record monocular gaze behavior, scene camera synchronized recording putter hit the ball the whole action, sampling frequency of 30 HZ.

Biofeedback Instrument: The Dutch Nexus-2 Biofeedback Instrument records ECG during putting in pressure situations from 6 s before to 1 s after club-ball contact^19^.

State Anxiety Inventory (S-AI): It was compiled by Spielberger et al. in 1970 and revised for the first time in 1979^20^, includes a total of 20 questions. The higher the score, the higher the degree of anxiety (Assessed by the 1–4 scale rating method). The test–retest reliability coefficient is 0.73–0.86 and the validity coefficient is 0.70–0.83.

**Figure 3:**
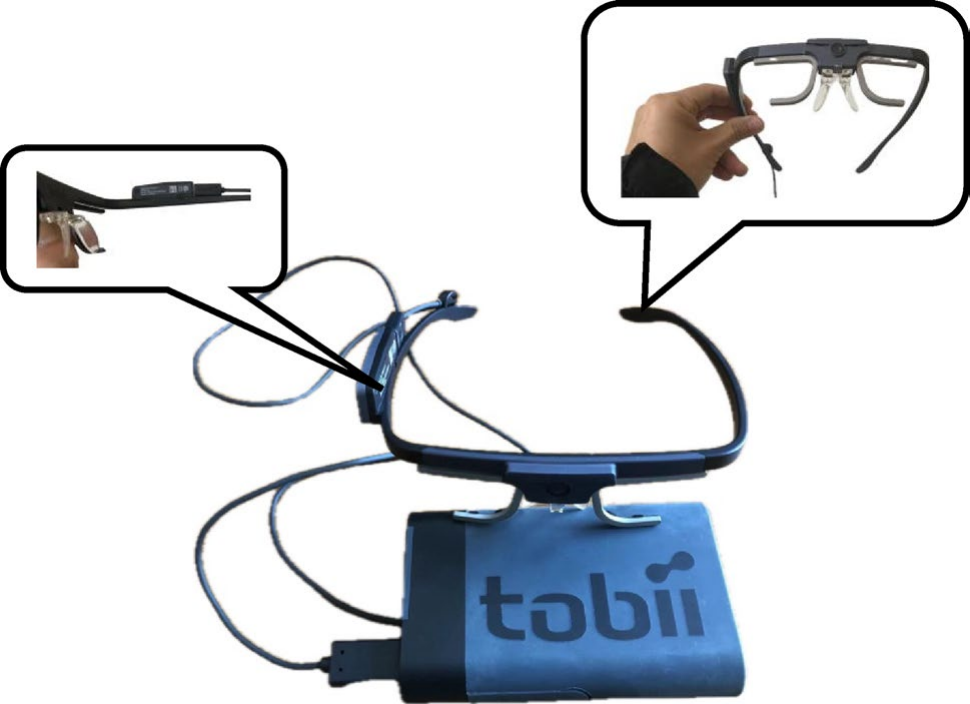
Diagram of experimental tools.

The experimental tools of this study are shown in Fig. 3.

### Data collection and statistical analysis

According to the movement characteristics of golf putts and previous research results, the entire putting process can be divided into four stages, namely the preparation stage (immediately after holding the ball and stabilizing it), the swing stage (immediately after the club starts to swing to touch the ball), the touch stage (immediately after the club touches the ball until the ball leaves the club), and the follow wave stage (when the ball leaves the club until the ball enters or does not enter the hole)^5,9^. After the experiment, the ASL built-in data analysis software was used to extract all fixation points for each participant during the putting process that had a fixation time exceeding 100 ms. The frame-by-frame analysis method was used to extract the fixation points, fixation time, and quiet eye movement time for each shot during each stage of the putting process. Based on previous research, this study defined quiet eye movement as the athlete's continuous gaze on the upper or back of the ball immediately before swinging the club backwards for more than 100 ms^5,9,21^, with the total fixation time being the sum of the fixation times at each stage.

SPSS 18.0 was used to analyze indicators such as hit rate, self-rated pressure level score, state anxiety score, heart rate, quiet eye movement time, total number of fixation points, and total fixation time, with a significance level of 0.05. If the repeated ANOVA of variance does not comply with the spherical assumption, the Greenhouse Geisser corrected *p*-value is taken.

## Results

### Comparison of hit rates

The hit rates (hit rate = number of holes/putts × 100) of participants when putting in pressure situations before and after training are shown in Table 1. Repeated measures ANOVA showed that the main effect of group was not significant, F (1, 20) = 2.115, *p* > 0.05; the main effect of test time was significant, F (1, 20) = 13.781, *p* < 0.05; and the interaction between group and test time was significant, F (1, 20) = 6.226, *p* < 0.05. Due to the significant interaction between groups and testing time, simple effect analysis showed that at the level of the quiet eye training group, the simple effect of test time was significant (*p* < 0.05); at the level of the technical guidance group, the simple effect of test time was not significant (*p* > 0.05); at the pre-training level and the post-training levels, the simple effects of group were not significant (*p* > 0.05). Compared with before training, the hit rate of the quiet eye training group was relatively higher after training, indicating that the accuracy of putting under pressure conditions improved after quiet eye training.

**Table 1:** Hit rate (M ± SD) during putting in pre/post-training pressure scenarios.

	Pre-training	Post-training
Quiet eye training group (%)	60.54 ± 4.59	65.18 ± 4.44
Technical guidance group (%)	59.90 ± 4.43	60.81 ± 4.09

### Comparison of self-assessed pressure levels

Before and after the training, when the participants put the putter in the pressure situation, they used the State Anxiety Scale (S-AI) to evaluate their pressure scores as shown in Table 2. Repeated measures ANOVA showed a significant main effect of group, F (1, 20) = 4.344, *p* = 0.050; a significant main effect of test time, F (1, 20) = 32.111, *p* < 0.05; and a significant interaction between group and test time, F (1, 20) = 18.778, *p* < 0.05. Because the group and test time interaction was significant, the simple effects analysis showed that at the level of the quiet eye training group, the simple effect of test time was significant (*p* < 0.05); at the level of the technical guidance group, the simple effect of test time was not significant (*p* > 0.05); at the pre-training level and the post-training levels, the simple effects of group were not significant (*p* > 0.05). Compared with before training, the self-assessed pressure scores of the quiet eye training group were relatively lower after training, indicating that the pressure felt when putting under pressure situations was reduced after the quiet eye training.

**Table 2:** Self-assessed pressure level scores (M ± SD) during putting in pre/post-training pressure scenarios.

	Pre-training	Post-training
Quiet eye training group	6.54 ± 0.82	5.18 ± 0.75
technical guidance group	6.63 ± 0.92	6.45 ± 0.82

### Comparison of state anxiety

The pre-training and post-training participants' state anxiety scores during putting in pressure situations are shown in Table 3. Repeated measures ANOVA showed a significant main effect of group, F (1, 20) = 7.994, *p* < 0.05; a significant main effect of test time, F (1, 20) = 25.489, *p* < 0.05; and a significant interaction between group and test time, F (1, 20) = 14.049, *p* < 0.05. Due to the significant group and test time interaction was significant, the simple effects analysis showed that at the level of the quiet eye training group, the simple effect of test time was significant (*p* < 0.05); at the level of the technical guidance group, the simple effect of test time was not significant (*p* > 0.05); at the pre-training level and the post-training levels, the simple effects of group were not significant (*p* > 0.05). Compared with before training, the state anxiety score of the quiet eye training group was relatively lower after training, indicating that the anxiety level when putting under pressure situations was reduced after quiet eye training.

**Table 3:** State anxiety scores (M ± SD) during putting in pre/post-training pressure scenarios.

	Pre-training	Post-training
Quiet eye training group	61.54 ± 6.50	51.09 ± 2.84
technical guidance group	62.36 ± 6.02	60.81 ± 4.57

### Comparison of heart rates

The heart rates of the participants during putting in pressure situations before and after training are shown in Table 4. Repeated-measures ANOVA showed that the main effect of group was not significant, F (1, 20) = 0.155, *p* > 0.05; the main effect of test time was not significant, F (1, 20) = 0.343, *p* > 0.05; and the interaction between group and test time was not significant, F (1, 20) = 4.206, *p* > 0.05. This indicated that there was no significant change in the heart rate of the pre-training and post-training. Participants heart rate did not change significantly when putting in pressure situations.

**Table 4:** Heart rate (M ± SD) during putting in pre/post-training pressure scenarios.

	Pre-training	Post-training
Quiet eye training group (times/min)	109.00 ± 8.42	110.36 ± 5.67
Technical guidance group (times/min)	111.91 ± 4.98	109.45 ± 4.69

### Comparison of gaze behavior

The quiet eye movement time, total number of fixation points, and total fixation time of the participants during putting in the pressure situation before and after training are shown in Table 5.

**Table 5:** Gaze behavior during putting in pre/post-training pressure scenarios (M ± SD).

Gaze behavior	Quiet eye training group		Technical guidance group	
	Pre-training	Post-training	Pre-training	Post-training
Quiet eye movement time (s)	1.83 ± 0.11	2.03 ± 0.12	1.82 ± 0.13	1.80 ± 0.05
Total number of fixation points (number)	18.09 ± 2.16	17.90 ± 2.02	18.09 ± 1.57	18.18 ± 1.72
Total fixation time (s)	7.45 ± 0.61	8.42 ± 0.64	7.43 ± 0.69	7.46 ± 0.47

Repeated-measures ANOVA on quiet eye movement time showed a significant main effect of group, F (1, 20) = 10.209, *p* < 0.05; a significant main effect of test time, F (1, 20) = 13.740, *p* < 0.05; and a significant interaction between group and test time, F (1, 20) = 17.730, *p* < 0.05. Due to the significant interaction between group and test time, simple effect analysis showed that at the level of the quiet eye training group, the simple effect of test time was significant (*p* < 0.05); at the level of the technical guidance group, the simple effect of test time was not significant (*p* > 0.05); at the pre-training level and the post-training levels, the simple effects of group were not significant (*p* > 0.05). Compared with before training, the quiet eye movement time of the quiet eye training group was longer after training, indicating that the quiet eye period became longer when putting under pressure after the quiet eye training.

A repeated-measures ANOVA on the total number of fixation points showed a non-significant main effect of group, F (1, 20) = 0.060, *p* > 0.05; a non-significant main effect of test time, F (1, 20) = 0.005, *p* > 0.05; and a non-significant interaction between group and test time, F (1, 20) = 0.055, *p* > 0.05. This indicated that there was no change in the number of total fixation points when participants putted in pressure situations before and after training.

A repeated-measures ANOVA on total gaze duration showed a significant main effect of group, F (1, 20) = 11.691, *p* < 0.05; a significant main effect of test time, F (1, 20) = 5.411, *p* < 0.05; and a significant interaction between group and test time, F (1, 20) = 4.651, *p* < 0.05. As a result of the interaction between group and test time was significant, simple effect analysis showed that at the level of the quiet eye training group, the simple effect of test time was significant (*p* < 0.05); at the level of the technical guidance group, the simple effect of test time was not significant (*p* > 0.05); at the pre-training level and the post-training levels, the simple effects of group were not significant (*p* > 0.05). Compared with before training, the total gaze time of the quiet eye training group was longer after training, indicating that the total gaze time when putting under pressure conditions became longer after quiet eye training.

## Discussion

In this study, the participants underwent 2 weeks of consecutive quiet eye training. Each day, they were required to complete three sets of putting exercises, consisting of 20 repetitions per set, following the instructions provided. The participants' putting process was assessed before and after the training under pressure situations using techniques such as eye movement recording, biofeedback, and subjective evaluation. The research findings revealed that the participants' putting accuracy improved after the quiet eye training in high-pressure situations. Additionally, their perceived pressure and state anxiety decreased. The duration of quiet eye movements and total fixation time increased, which supports the hypothesis of the study.

This study stimulated pressure with reference to current common methods of conducting group putt-test competitions with the added effects of payoffs (winning group receives a ￥2000 team reward) and video cameras (videotaping the testing process and feeding the results back to the coaching staff). Direct competition can increase the motivation for success, the likelihood of winning a payoff increases perceptions of importance, and the role of video camera is similar to that of a spectator, which can enhance situational perception of pressure and is also a commonly used pressure manipulation method in experiments^19,21^. The results of the study showed that after two consecutive weeks of quiet eye training participants had increased hit rates and decreased pressure perceptions and state anxiety when putting in pressure situations. Wood et al.^22^ found similar results in the study of quiet eye training for soccer penalties^19^. Fulton et al.^23^ used the golf putting task to explore the correlation between action performance and quiet eye movements, and found that there was a significant positive correlation between action performance and quiet eye movement duration. Vine et al. (2014) concluded that quiet eye training is an effective method of counteracting anxiety^8^. The reason why quiet eye training can reduce anxiety or competition pressure, on the one hand, may be because it can help athletes better understand when and where to focus on various aspects of action execution, increase individual information processing efficiency^24^, reducing the interference of irrelevant information^4,23,25^; on the other hand, it may be due to the increased sense of subjective control of athletes^26^, due to its ability to enable athletes to adopt reasonable gaze strategies, which enhances their perceptions of their own coping resources, their cognitive evaluation of pressure situations becomes more positive (i.e. perceived as a challenge rather than a threat)^24,27^. The increase in the sense of control is closely related to positive cognitive evaluation and control of anxiety levels^28^.

The availability of lightweight portable eye-trackers has facilitated researchers to explore attentional behaviors of visually dominant movement skills in a more natural setting. The present study used portable eye-trackers to test participants' gaze behaviors during putting in a pressure situation and found that their quiet eye movement duration and total gaze duration increased after two consecutive weeks of quiet eye training. Wood et al.^22^, Vine and Wilson^13^ also found similar results in the studies of quiet eye training in which participants' quiet eye periods were prolonged after quiet eye training^13,19^. Chia et al.^6^ argued that the quiet eye before the movement begins is an important feature of visual search strategies, which ensures that athletes have sufficient time to focus on task-relevant cues due to the dynamic complexity of sports situation, and that prolonged gaze can help the movement performer to filter the key information before completing their skills^4,29^. The increase in total fixation time may be related to the increase in quiet eye movement time^30^, i.e., caused by an increase in the duration of sustained gaze on the upper or back of the ball before the athlete immediately swings the club backward. Quiet eye training is the final ocular fixation that precedes critical athletic movements and that enables athletes to gather relevant information and organize their subsequent movement^31–33^. As the quiet eye movement time and total fixation time become longer, the pressure sensitivity and state anxiety of athletes in the quiet eye training group were reduced, and golfers' hit rate when putting under pressure increased. This research result is consistent with the findings of Broodryk et al.^34^, especially under high pressure, short quiet eye training is beneficial the attention control and accurate goalkeeping performance of rugby goalkeepers, and quiet eye during three-point shots in basketball could fulfill an online control function^35^.

In conclusion, after the implementation of two consecutive weeks of quiet-eye training in this study, the participants were able to improve their hit rate when putting in pressure situations, and the reason why the quiet-eye training was able to improve their putting performance was related to the attentional strategies they used. The prolongation of the quiet eye period after the training not only helped in the acquisition and processing of key information cues, but also served to reduce anxiety to a certain extent. The results of this study provide a rationale and methodology for attentional training and control of putting in golfers, as well as research ideas for the psychological regulation of competition in closed sports that require visual dominance.

Despite the interesting findings from the present study, the following limitations should be kept in mind. First, although we used commonly used methods to simulate pressure situations, it remains uncertain whether the performance observed after the quiet eye training intervention transfers to real-world competition^36,37^. Therefore, in the future research should attempt to collect data from real-world competitions, especially statistics of key competitions, to determine the transfer effects of quiet eye training interventions more accurately. Second, given individual variability, further research should differentiate between the effects of quiet eye training interventions on everyone.

## Conclusion

This study conducted two consecutive weeks of quiet eye training, and used eye-tracker, biofeedback instrument and state anxiety inventory (S-AI) to test and analyze the participants' putting process under pressure situations before and after training. The results of the study show that quiet eye training can improve golfers' putting performance under pressure situations. The duration of quiet eye training, state anxiety decreased, quiet eye movements and total fixation time increased.

From an application perspective, the results suggest that quiet eye training can be used to improve attentional control in golfers and potentially minimize the adverse effects of anxiety on performance in high-pressure situations. However, further research should try to explore the relationship between attention strategies, interference conditions, etc. and quiet eye and putting performance to provide a basis for targeted attention training.

### Supplementary Information

Supplementary Information.

## Data Availability

Some of the data for this study can be found in Supplementary File “Data of the Study”, and the rest can be obtained by contacting the corresponding author.
